# Motherhood in breast cancer survivors: Challenges and opportunities

**DOI:** 10.22088/cjim.15.2.367

**Published:** 2024

**Authors:** Marzieh Azizi, Elham Ebrahimi, Zahra Behboodi Moghadam, Zohreh Shahhosseini, Behjat Khorsandi, Maryam Modarres

**Affiliations:** 1 Department of Midwifery, School of Nursing and Midwifery, Sexual and Reproductive Health Research Center, Mazandaran University of Medical Sciences, Sari, Mazandaran, Iran; 2 Department of Midwifery and Reproductive Health, School of Nursing & Midwifery, Tehran University of Medical Sciences, Tehran, Iran; 3 Research Center for Nursing and Midwifery Care, Shahid Sadoughi University of Medical Sciences, Yazd, Iran; 4 Department of Midwifery and Reproductive Health, Nursing and Midwifery Care Research Center, School of Nursing & Midwifery, Tehran University of Medical Sciences, Tehran, Iran

**Keywords:** Breast cancer, Survivors, Motherhood

**Dear Editor**

Breast cancer (BC) is one of the most commonly diagnosed cancers among reproductive-aged women worldwide (1) and is responsible for nearly 30% of all cancers that occurs in these women (2). An increasing incidence of BC among young women aged<40 and also a growing pattern of delay in childbearing decision-making among couples in developed and also developing countries (3) due to various reasons such as socioeconomic, educational, professional, and infertility history leads to considerable concerns regarding the fertility issues for young BC survivors without children (4). 

Factors including the possible effect of adjuvant therapy on women’s fertility and the need for long-term hormone therapy consumption were the main reasons for the lower pregnancy rate among BC survivors (5). Studies indicated that BC treatment might reduce the possibility of fertility by inducing premature ovarian failure and induced menopause (2, 4), so fertility preservation methods have attracted substantial attention among physicians and are considered an essential part of cancer care (6, 7). In recent decades, the development of new fertility preservation techniques and consideration of fertility counseling for reproductive-aged women increased the young survivor’s intention to conceive after treatment and experience motherhood (8). As reproductive health specialists, our research team performed a qualitative study regarding pregnancy experience and perceived needs through the lens of BC survivors. According to the results, we understood that their concerns regarding their fertility issues after BC diagnosis and treatment were essential. We designed an educational package for BC survivors regarding their pregnancy health. As interested researchers in BC, we wrote a letter to the editor to answer the question, "Which challenges and opportunities do *BC survivors face regarding motherhood after the treatment of BC?” *

There are different ideas regarding the experience of motherhood among BC survivors. Some women declared motherhood after cancer was a miracle of God and an unexpected pleasant event (9). Also, experiencing motherhood leads to higher self-esteem, the sense of being stronger to cope with challenges, higher energy levels to perform daily activities, and higher psychological well-being (9, 10). BC survivors also declared that they have a sense of a healthy and robust mother, find an incredible opportunity to return to everyday life before a cancer diagnosis, and fill up all of their sadness and hopelessness of their previous thoughts regarding their inability to childbearing during treatment (9). 

Against these positive aspects of motherhood, the transition to motherhood responsibilities accompanied by the fear of cancer recurrence and becoming an ill mother are some of the challenges among survivors (11). Many women have considerable anxiety and distress regarding their ability to complete a healthy pregnancy. They also fear the possible adverse effect of adjuvant therapy on their fetus's health and development, such as congenital abnormalities (5). Concerns regarding transmitting the genes of cancer to newborns (5) and fear of losing a pregnancy and its subsequent grief were other BC survivors' challenges (7, 12). Regarding the effects of pregnancy on cancer recurrence, most studies showed that pregnancy did not increase the risk of relapse among BC survivors (13, 14). However, there is no specific evidence about the appropriate time of pregnancy after completion of BC treatment. Women with estrogen receptor-negative BCs who became pregnant and gave birth within two years after the cancer treatment showed no disease recurrence (1, 12). In addition, maternal and neonatal health complications did not significantly increase among BC survivors compared to the general population (13, 15, 16).

Fear of losing the ability to breastfeed due to mastectomy, fear of inability to devote enough energy to taking care of the newborn and meeting the infant or other child’s needs (17), and the sense of dependency on family members, especially the husband’s support to perform motherhood responsibilities (10) were the most common challenges of BC survivors after childbirth. Also, the fear of motherless children growing up and concern regarding the child’s well-being after the mother's death was reported by some of the BC survivors with experience of motherhood after treatment (9, 10). Safety and feasible breastfeeding among BC survivors is an important issue in motherhood, so they need essential supportive counseling regarding the ability to breastfeed during prenatal and postnatal care (18). The results showed that the milk production in one breast would occur in BC survivors with unilateral mastectomy and are sufficient for neonates’ nutritional needs, so mothers should be encouraged to initiate and continue breastfeeding after childbirth (5). To our knowledge, motherhood is a pleasant inseparable part of women’s lives, and each woman, even BC survivors, hopes to experience this unique event positively. The concerns mentioned by BC survivors regarding the motherhood experience were due to the limited information received from healthcare providers (19).

Despite motherhood in BC survivors are associated with negative attitudes and fear toward death and breastfeeding, even though the presence of a child significantly increases the hope level and quality of life among new mothers (9). The authors of this study suggest that to improve the happiness and psychological well-being among these women regarding motherhood, consider the multidimensional supportive program and survivorship care after treatment regarding fertility issues proposed. This program can include the possibility of a healthy pregnancy and childbirth, safe breastfeeding, a high survival rate after pregnancy, and the ability to grow their newborn, leading to converting motherhood as an opportunity and hope for them after experiencing a complicated treatment process. 
